# Role of Solvent Polarity in the Extraction and Electrochemical Behavior of Antioxidants From *Ziziphus jujuba* Leaves

**DOI:** 10.1155/ianc/7600950

**Published:** 2026-11-10

**Authors:** Umme Kalsoom, Zahid Hussain, Salman Khan, Naveed Ahmad, Shohreh Azizi, Malik Maaza

**Affiliations:** ^1^ Department of Chemistry Abdul Wali Khan University Mardan Mardan 23200 Pakistan awkum.edu.pk; ^2^ UNESCO-UNISA Africa Chair in Nanosciences/Nanotechnology College of Graduate Studies University of South Africa Pretoria 0181 South Africa unisa.ac.za; ^3^ Department of Chemical and Materials Engineering College of Engineering Northern Border University Arar Saudi Arabia nbu.edu.sa

**Keywords:** cyclic voltammetry, flavonoids, LC-MS, redox activities, saponins, solvent polarity, *Ziziphus jujuba*

## Abstract

*Ziziphus jujuba* is a medicinal plant known for its rich content of antioxidants including flavonoids, saponins, phenolics, and triterpenoids. This study determines the effect of solvent polarity on the extraction yield and investigates phytochemical distribution of the extracts and its electrochemical behavior. Reflux extraction was used for the preparation of crude extracts, using methanol, ethanol, and water. The ethanolic extract was selected for solvent fractionation, using n‐hexane, dichloromethane, chloroform, and ethyl acetate. Among the extraction solvents, ethanol produced the highest crude yield (6.0% w/w). Following solvent–solvent fractionation, the ethyl acetate fraction showed the highest recovery among all fractions (0.30% w/w). The presence of flavonoid and saponin fractions was confirmed by phytochemical screening. The highest absorbance was observed in ethyl acetate fraction through UV visible spectrophotometric analysis, *A* = 0.711 for flavonoids at 415 nm and 0.330 for saponin at 406 nm. Cyclic voltammetry (CV) for ethyl acetate and chloroform fractions showed oxidation peaks at 0.50 V (vs. Ag/AgCl). Fourier transformed infrared spectroscopy (FT‐IR) analyzed functional groups of flavonoids and saponins. Liquid chromatography mass spectrometry (LC‐MS) identified major flavonoids, that is, vicenin II, spinosin, kaemferol (glycoside), and saponins (jujubosides). The result stated that the polarity of solvents plays a vital role in extracting redox‐active antioxidants from the leaves of *Z. jujuba.* Furthermore, it stated that ethyl acetate is the most effective fractionation solvent. This study gives analytical framework for extraction and characterization of antioxidant‐rich fraction of *Z. jujuba* leaves along with emphasis on the role of solvent polarity in phytochemical recovery.

## 1. Introduction

Antioxidants are known to neutralize free radicals and alleviate oxidative stress, protect cellular structures against damage, and prevent lipid peroxidation, stabilizing reactive oxygen species, as well as promoting overall biochemical balance in living systems. Their presence leads to an enhanced immune system, slows down aging, and lowersthe chances of chronic diseases like cardiovascular disorders, diabetes, and some types of cancer. They are abundantly present in fruit and vegetables of angiosperms and in grains, nuts, legumes, teas, and a broad variety of medicinal medical plants [1]. Phytochemical constituents are common in medicinal plants. Among the medicinal plants, *Ziziphus jujuba* is especially known to possess an extensive antioxidant profile, which has a long‐established therapeutic potential. It is a perennial fruit tree in the Rhamnaceae family with over 3000 years of use in traditional medicine [2]. According to these surveys, there were 4.3–5.4 thousand hectares of *jujube* and ber orchards in Pakistan with 22–28 thousand tons of fruits per year. Given its antioxidant richness and wide availability, *Z. jujuba* has become an important candidate for antioxidants, ranging from highly polar phenolic acids and flavonoids to less polar aglycones, triterpenoids, and saponins. Due to the complicated composition of polar and nonpolar components, extraction and fractionation of *jujuba* frequently employed a combination of several extraction methods and solvents separation to optimize the percentage yield of the crude and yield of the desired antioxidants [3]. Several conventional and modern techniques were used to obtain crude with higher % yield, including maceration, reflux extraction, and microwave‐assisted extraction (MAE) [4]. Among these, reflux extraction is widely regarded as the most efficient, due to maintenance of a controlled temperature near the solvent boiling point, enhances mass transfer, improves solvent contact with the plant matrix, and prevents solvent evaporation. For instance, Soib et al. compared different extraction methods on *Carica papaya* leaves and reported that reflux extraction produced the highest crude yield (17.86%) compared to ultrasonic‐assisted extraction (13.57%) under the same conditions [5]. Similarly, Faktorial compared multiple extraction techniques on *Lannea coromandelica* leaves and found that ethanolic reflux produced the highest yield (30.8%), whereas maceration yielded 18.7% [6]. Nevertheless, despite the potential thermal sensitivity of certain phenolic and glycosidic compounds, reflux extraction had been continuously widely applied in phytochemical investigation, owing to its ability to achieve higher extraction recoveries under controlled conditions. Following crude extraction, the extract was typically subjected to liquid–liquid fractionation, where it was partitioned sequentially into solvent of increasing polarity, that is, n‐hexane < chloroform < ethyl acetate < butanol < water. This method effectively separates complex phytochemical mixtures based on polarity, resulting in flavonoids, phenolic acids, or saponins [4]. To efficiently recover antioxidant phytochemicals from plant matrices, the choice of extraction solvent is critically dependent on solvent polarity. For instance, aqueous ethanol (50%–80%, v/v) is widely regarded as the most effective solvent system for extracting phenolics and flavonoids [7]. Despite the effectiveness of aqueous ethanol for maximizing phenolic recovery, pure solvents remain a valuable analytical approach for selective extraction and phytochemical partitioning. Furthermore, a solvent with medium polarity, such as ethyl acetate, is frequently used to obtain semi‐polar phenolics during fractionation, whereas nonpolar solvents, that is, n‐hexane, selectively extract lipophilic constituents, including carotenoids and pentacyclic triterpenoids. Saponins, being amphiphilic glycosides composed of a hydrophobic aglycone linked to hydrophilic sugar chains, typically partition into medium to high polarity solvents; however, certain structural variants may also appear in moderate polar solvents such as chloroform. Ramdani et al. extracted flavonoid content from *Calicotome spinosa* leaves using various solvents through liquid–liquid fractionation method, the highest flavonoid contents were obtained in ethyl acetate (20.87 mg QE g^−1^), followed by the n‐butanol fraction (17.03 mg QE g^−1^) [8]. Characterizing these antioxidants is essential for understanding their chemical nature, redox properties, and biological potential, which requires the use of advanced analytical techniques. Among these, cyclic voltammetry (CV) and LC‐MS are widely used through the measurement of oxidation–reduction responses. CV offers quick electrochemical screening, indicating the existence and relative effectiveness of electroactive antioxidants. Furthermore, LC‐MS provideshigh‐resolution molecular profiling, which makes it possible to separate, identify, and detect a wide range of antioxidants using chromatographic retention patterns and mass‐to‐charge ratios. For example, Bora et al. applied CV to *Swietenia macrophylla* bark extract and found oxidation maxima at 0.1 V for flavonoid and 0.17 V for phenolic [9]. In a different investigation, Kao et al. used HPLC‐MS to identify 34 saponins and 8 flavonoids in methanol extract of *Gynostemma pentaphyllum* [10]. However, majority of the studies focused on extraction under a single extraction condition. Additionally, LC‐MS profiling of *Ziziphus* leaves, seeds, and fruits is well established. While comparative evaluations across solvent polarities and integrated electrochemical assays remain limited. This study addresses this gap by systematically varying solvent extraction, thus compound polarity, and combining extraction yield measurements with TLC, FT‐IR, UV‐vis spectrophotometry, CV, and LC‐MS to assess how solvent choice affects antioxidant recovery and redox behavior. UV‐visible spectrophotometry is used for semiquantitative estimation and separate content, while thin‐layer chromatography (TLC) is used for metabolite profiles and relative polarity‐based separation. Structure features and functional groups are characterized by FT‐IR spectroscopy, and major phytochemicals are identified using LC‐MS analysis. In addition, the antioxidants capacity and redox behavior of the extracts are investigated through CV to elucidate the electron transfer characteristics. The result provides solvent dependent antioxidants recovery and establishes a framework for the extraction and characterization of antioxidants in *Z. jujuba* leaves.

## 2. Experimental

### 2.1. Plant Material

Leaves of *Z. jujuba* were collected from Katlang Hills, Mardan, Khyber Pakhtunkhwa, Pakistan (34.377 N, 72.017 E). The sample was identified by Dr. Mohib Shah, Department of Botany, Abdul Wali Khan University, Mardan, Pakistan.

### 2.2. Reagents and Chemicals

All solvents and reagents used in this study were of analytical grade. Ethanol (95% purity), methanol (≥ 95% purity), and distilled water were used as extraction solvents. Solvent–solvent partitioning employed n‐hexane (≥ 95% purity), dichloromethane (≥ 97% purity), ethyl acetate (≥ 98% purity), and chloroform (≥ 99% purity). Analytical reagents including ferric chloride (≥ 98% purity), hydrochloric acid (HCl) (1 M), aluminum chloride (AlCl_3_) (2% aqueous), and sulfuric acid (H_2_SO_4_) were procured from Merck and Sigma‐Aldrich. All chemicals were used without further purification.

### 2.3. Instrumentation

The extracts were concentrated under reduced pressure using a rotary evaporator (Buchi, Switzerland). A double beam UV‐visible spectrophotometer was used to record UV absorbance. The FT‐IR spectra were recorded 4000–400 cm^−1^ in a FT‐IR spectrometer. The electrochemical measurements were performed using CV with a potentiostat, a glassy carbon working electrode, Ag/AgCl reference electrode, and platinum counter electrode. LC‐MS/MS was analyzed by a Shimadzu prominence UFLC system combined with a LC‐MS‐8040 triple quadrupole mass spectrometer.

### 2.4. Sample Preparation

Freshly collected *Z. jujuba* leaves were washed with distilled water to remove dust and surface contaminants and air‐dried under a fume hood over 48 h at ambient temperature, and ground to fine powder using a mechanical grinder. The biomass was dried and kept in airtight containers awaiting further analysis.

### 2.5. Extraction of Crude Extracts

Reflux extraction was used to prepare crude extracts. Leaf powder of 50 g was combined with 250 mL of ethanol, methanol, or distilled water in every extraction. The mixture was refluxed in 2 h and then filtered on filter paper. A rotary evaporator was used to concentrate the filtrations at a reduced pressure to produce crude. All extraction experiments were performed three times, and the extraction yields are reported as mean ± standard deviation (SD).

### 2.6. Solvent–Solvent Fractionation

A 10‐g portion of the ethanol extract was dissolved in distilled water to form an aqueous solution which was sequentially fluctuated using 100 mL each of n‐hexane, dichloromethane, chloroform, and ethyl acetate, following increasing solvent polarity. Each extraction step was repeated in triplicate to ensure complete separation of the chemical constituents. Organic layers were collected, dried under reduced pressure, and stored at 4°C, the solvent–solvent fractionation scheme is illustrated in Figure 1.

**FIGURE 1:**
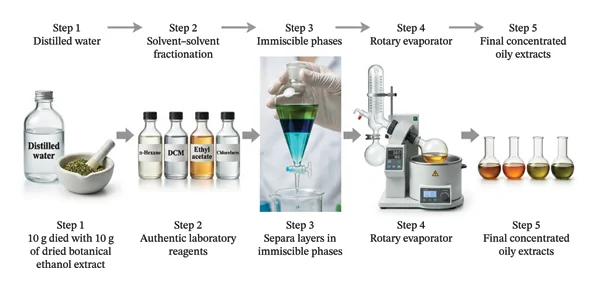
Liquid–liquid fractionation using various solvent systems including n‐hexane, DCM, ethyl acetate, and chloroform.

### 2.7. Phytochemical Screening (Spot Test)

The extracts were screened for saponins and flavonoids using standard calorimetric spot tests. In the case of saponins, ethanol:sulfuric acid (9:1, v/v) was used in each extract. A characteristic violet coloration which changed to the original green color of the extract indicated the presence of saponins. In the case of flavonoids, 2% aqueous AlCl_3_ solution was added in the extract. The color changes from green to yellow to confirm the formation of flavonoid–AlCl_3_ complex.

### 2.8. Thin‐Layer Chromatography

TLC was performed on silica gel plates and DCM:methanol (1:1) was used as mobile phase. Solvent fractions were applied as small spots using a capillary tube. The TLC plates were air‐dried and examined under ultraviolet (UV) light. The plates were sprayed with 2% AlCl_3_ solution for flavonoids and ethanol:sulfuric acid (9:1, v/v) for saponins detection. The retention factor (Rf) values were calculated as the ratio of distance traveled by each compound to the distance traveled by the solvent front.

### 2.9. Characterization

UV‐visible spectrophotometry was conducted by recording absorbance at the respective *λ*, that is, 415 nm (flavonoids) and 460 nm (saponins). FT‐IR was carried out to determine the functional groups that exist in each fraction. Samples were directly deposited on the attenuated total reflectance (ATR) crystal, and a spectrum was acquired at 4000 to 400 cm^−1^ as 4 cm^−1^. The electrochemical properties of the solid fractions were elevated using CV. Measurements were conducted in the potential window of 0.0 to +1.0 V using a glassy carbon as working electrode, Ag/AgCl as reference electrode, platinum wire as counter electrode, 0.1 M KCl solution was used as the supporting electrolyte, and fractions were dissolved in distilled water. All voltammograms were recorded at a scan rate of 100 mV s^−1^. The LC‐MS analysis was run on Shimadzu UFLC with an LC‐MS‐8040 Triple quadrupole system mass spectrometer equipped with electrospray ionization source. The column was separated on a Shim‐pack XR ODS III column (150 × 3.0 mm, 2.2 μm) held at 40°C. The mobile phase consists of solvent A (0.1% formic acid in water) and solvent B (acetonitrile, HPLC grade). Gradient elusion was performed as follows: 0–2 min, 5% B; 2–17 min, 5%–30% B; 17–20 min, 30%–55% B; 20–35 min, 55%–100% B; 35‐37 min, 100%–5% B; and 37–39 min, 5% B for column re‐equilibrium. The flow rate was maintained at 0.3 mL/min, the injection volume was 10 μL. Electrospray ionization (ESI) was set in the positive/negative ion modes (depending on analytical conditions). Phytochemical constituents were determined according to the retention times (RT), molecular ion peaks, and the fragmentation patterns of the MS/MS and were compared with the published literature data to tentatively identify compounds.

## 3. Result and Discussion

### 3.1. Effect of Solvent on *Z. jujuba* Crude Yield and Optimization

Extraction yield depends on solvent polarity, as plant metabolites are released only when the solvent can effectively interact with them through polarity and hydrogen bonding [11]. In this study, three different solvents, ethanol, methanol, and distilled water were used for crude extraction from *Z. jujuba* leaves. The result indicated variation in the extraction yield, demonstrating polarity‐dependent behavior of *Z. jujuba*. Among them, ethanol gave the highest yield of crude extract (3.00 ± 0.04 g; 6.00 ± 0.07% w/w), followed by methanol (2.70 ± 0.02 g; 5.40 ± 0.04% w/w) and water (2.0 g) (2.00 ± 0.02 g; 4.00 ± 0.04% w/w). The superior performance in ethanol was due to its intermediate polarity (polarity index = 5.2), which makes it capable of dissolving both the polar components (e.g., phenolics, saponins, sugars) and moderately nonpolar components (e.g., flavonoid aglycones, triterpenoids). On the other hand, however, methanol is more polar than ethanol, resulted in less % yield, a similar preference for ethanol over methanol was also observed in the leaf extracts of *Ziziphus mauritiana* [12], which could be attributed to the excessive polarity, which may reduce the solubility of moderately lipophilic metabolites. The lowest % crude extract was observed in water, when used as solvent, reflecting selective extraction of highly polar compounds, while strong hydrogen‐bonding network restricted its ability to dissolve midpolarity molecules [13]. Water exhibits the weakest penetration and lowest solvation capacity for semipolar molecules, consistent with lower crude yields in extractions. The effect of solvent polarity on crude extract yield is presented in Figure 2a.

**FIGURE 2:**
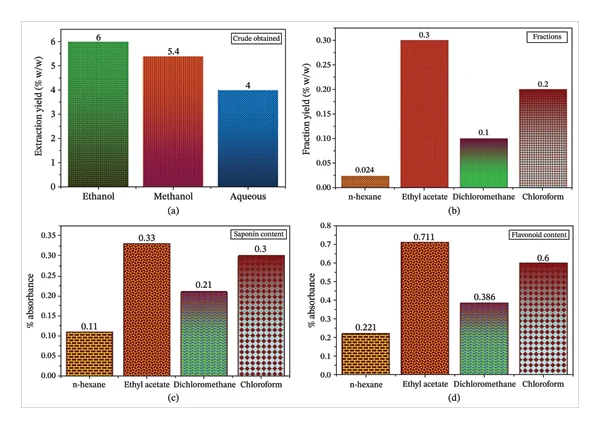
(a) Ethanol, methanol, and water yields of crude extract as a percentage. (b) Distribution of extraction yield through solvent fractions, that is, n‐hexane, DCM, chloroform, and ethyl acetate. (c) Flavonoid (absorbance at particular wavelength) in each solvent fraction. (d) Saponin content (absorbance at a particular wavelength) at various solvent fractions.

### 3.2. Foam Test

Several preliminary tests are used for the detection of antioxidants in plant extracts, provide the evidence of bioactive compounds based on their chemical properties, such as amphiphilic nature, surface activity, and glycosidic structure, and interactions with solvents and air molecules. These tests serve as an initial qualitative assessment and help in correlating physicochemical behavior with the presence of specific secondary metabolites [14, 15]. Among them, foam tests use the amphiphilic behavior of bioactive compounds, where the ability to form and stabilize foam is directly associated with surface‐active constituents. In this study, saponins were detected in the ethanolic extract of *Z. jujuba* using a foam test. The result indicated that, after vigorous shaking, a stable froth of 2 cm in height was obtained that remained for 10 min. This indicated the presence of saponins, which are amphiphilic glycosides [14], that lower surface tension and produce persistent foam due to their hydrophilic sugar moiety and hydrophobic aglycone structure [15]. Similar other work reported the same results. For instance, Góral and Wojciechowski studied the surface activity and foaming properties of saponin‐rich plant extracts, they performed the foam tests of 45 plant extracts, and the result indicated that the foam obtained remained stable for at least 10 min [14].

### 3.3. Spot Test

The spot test identifies saponins and flavonoids with the help of the reagents that react with structural characteristics, such as glycosidic bonds, aromatic rings, and hydroxyl groups, resulting in typical color changes. The spot test in the current research was carried on TLC plate with ethanol:sulfuric acid (9:1, v/v) as the saponins reagent and 2% aqueous AlCl_3_ as the flavonoids reagent. The result for the saponin test indicated the changing of green to violet color, validating the existence of saponins, is due to inducing conversion of triterpenoid sapogenins into conjugated carbocation intermediates [16]. These intermediates subsequently condense into violet–purple polymeric derivatives [17]. Similarly, for the flavonoid, the color changed from yellow to greenish, this could be due to the formation of Al^3+^ flavonoid complexes, AlCl_3_ chelates with *ortho*‐dihydroxyl (catechol) groups or 3‐ and 5‐hydroxy‐4 keto systems commonly present in flavones and flavanols. This chelation increases conjugation within the chromophore, resulting in a pronounced yellow‐to‐green fluorescence. Our findings are similar with previous work for saponin reported by Cong‐Hau et al. [18].

### 3.4. Distribution of Extraction Yield Across Various Solvent Fractions

The solvent extraction primarily depends on physicochemical compatibility between the solvent and plant metabolites [13, 19], for example, solvent polarity, dielectric constant, and hydrogen‐bonding capacity. Particularly the solvents that match the polarity of the target constituent extract greater quantities of the desired bioactive. This higher extraction is ascribed to effective disruption of solute interactions and facilitate mass transfer into the solvent phase [4, 11, 19]. The solvents used in the current work included four solvents namely ethyl acetate, chloroform, DCM, and n‐hexane. The outcome showed that the ethyl acetate, chloroform, DCM, and n‐hexane produced 0.030 g (0.30% w/w), 0.020 g (0.20% w/w), 0.010 g (0.10% w/w), and 0.0024 g (0.024% w/w), respectively. The observed highest extraction in ethyl acetate is due to intermediate polarity index and favorable hydrogen‐bonding characteristics, enabling solubilization of a broad spectrum of moderate polar phytochemicals such as phenolics, triterpenoids, and glycosides [13, 20]. Chloroform, being less polar than ethyl acetate, its high polarizability and ability to stabilize induced dipoles allow it to dissolve semipolar constituents [4, 19], yielded 0.020 g (0.20% w/w). In the case of DCM, it resulted in lower mass extract, that is, 0.010 g (0.10% w/w). It has lower dielectric constant and weaker solvation ability, limiting its ability to disrupt plant matrix interactions. Among all fractions, the lowest extraction yield was observed for the n‐hexane fraction, which can be attributed to its highly nonpolar nature and inability to engage in hydrogen bonding or dipole–dipole interactions. As a result, n‐hexane selectively extract only a limited lipophilic constituents, such as waxes and nonpolar terpenes, while excluding the majority of polar and semipolar phytochemicals present in plant matrix, yielding a minimal extractable mass [19]. The distribution of extraction yield across the different solvent fractions is shown in Figure 2b. Comparable observations showing higher yields in ethyl acetate fractions have been reported by Chatepa et al. and Thakur et al. who similarly found that ethyl acetate extracts significantly higher amounts of phytochemicals than chloroform, DCM, or hexane [21, 22].

### 3.5. Solvent Effect on Flavonoid and Saponin Content

UV‐visible spectrophotometry quantifies flavonoids and saponins by measuring light absorption resulting from their intrinsic chromophores formed with specific reagents. Beer–Lambert law correlates observance directly with solute concentration [23]. It is used to compare extraction efficiency across solvents. The results showed that ethyl acetate yielded the highest absorbance for both flavonoids (0.711) and saponins (0.330), followed by chloroform (0.600 for flavonoids and 0.300 for saponins), then DCM (0.386 and 0.210) and n‐hexane (0.221 and 0.110). The extraction of solvents for these compounds increased in the order n‐hexane < DCM < chloroform < ethyl acetate. The greater extraction ability of ethyl acetate is due to its intermediate polarity (polarity index = 4.4) and strong hydrogen bond capacity, which help to dissolve moderately polar phytochemicals like flavonoids and saponins that contain phenolic hydroxyl and glycosidic groups [11, 23]. The effectiveness of ethyl acetate in extracting antioxidants is also reported in *Ziziphus lotus* [24]. Chloroform yielded the second highest value, though less polar than ethyl acetate, its high polarizability and ability to stabilize induced dipole–dipole and van der Waals interactions allow it to extract semipolar constituents such as flavonoid and triterpenoids [4]. In contrast, DCM produce only moderate observance values for both compound classes, because of its lower dielectric constant and weaker solvation efficiency, limits its ability to disrupt the strong matrix interaction of plant, holding phenolic and glycosylated compounds in the plant material [25]. These findings agree with Mohebatti et al. who similarly reported that ethyl acetate fractions consistently yielded the highest flavonoids and saponins concentration among organic solvents, confirming the same extraction order observed in this study [26]. Similar solvent‐dependent reductions in flavonoid recovery with DCM were reported by Bui et al. who observed that DCM consistently yields less than ethyl acetate or chloroform fractions [27]. The solvent‐dependent variation in flavonoid and saponin absorbance values is illustrated in Figure 2c,d, respectively.

### 3.6. TLC of Solvent Fractions of Leaves

TLC is used for preliminary identification of plant secondary metabolites, including flavonoids and saponins. Separation of these bioactive compounds is based on a stationary phase, that is, silica gel, differences in compound polarity, and affinity toward the mobile phase, resulting in characteristic migration distances expressed as Rf values [28]. In this study, TLC analysis of solvents fractions of *Z. jujuba* leaves was performed using a DCM:methanol (1:1) solid system. This was selected for intermediate polarity, which facilitate effective separation of both semipolar and moderately polar phytochemicals [29]. DCM facilitates the migration of less polar constituents, whereas the methanol enhances the elution of polar compounds such as flavonoids and saponins through hydrogen‐bonding interactions, resulting in improved band resolution on silica gel plates. The TLC analysis revealed solvent‐dependent variations in phytochemical composition. The n‐hexane column showed three different bands with Rf values of 0.224–0.88. The dichloromethane fraction had five bands with Rf values between 0.18 and 0.83. Six bands with Rf values between 0.20 and 0.87 were obtained with the chloroform fraction. The ethyl acetate fraction had the richest chromatographic profile as it gave seven well‐resolved bands with Rf value between 0.16 and 0.90. Increasing number of TLC bands observed from n‐hexane to ethyl acetate quantitatively confirms the strong influence of solvent polarity on phytochemical extraction and separation [29]. These findings are in close agreement with the previous studies reporting superior TLC resolution and higher photochemical diversity in ethyl acetate fraction of medical plant leaves, particularly for flavonoids and saponins [30].

### 3.7. Cyclical Voltametric Analysis of *Z. jujuba* Solvent Fractions

The redox behavior of solvent fractions obtained from *Z. jujuba* leaves was investigated using CV to evaluate the distribution of electroactive antioxidant across solvents of varying polarity [31, 32]. CV is a well‐established electroanalytical technique for investigating phenolic compounds and flavonoids, which typically undergo reversible or quasi reversible oxidation within the potential range of 0.2–0.8 V versus Ag/AgCl [31]. In this study, distant anodic oxidation peak was observed for the ethyl acetate and chloroform fractions at approximately Epa = 0.50 V (Figure 3a), confirming the presence of electroactive antioxidant compounds in these midpolarity extracts. However, well‐defined anodic peaks were detected in the n‐hexane or DCM fractions, indicating the absence or extremely low absorbance of redox active phenolic and flavonoids in these less polar solvents. Quantitatively, ethyl acetate fraction exhibited the highest anodic peak current Ipa = 1.5 × 10^−5^ A, followed by the chloroform fraction Ipa = 7.5 × 10^−6^ A. Although both fractions displayed an identical oxidation potential at 0.50 V, the higher Ipa recorded for ethyl acetate reflects a greater concentration of oxidizable antioxidant. The observed peak potential is characteristics of catechol type phenolics and flavonoids and is consistent with oxidation profiles reported for polyphenol‐rich plant extract [31, 33]. The absence of any significant anodic response in the n‐hexane fraction is expected as highly nonpolar solvents primarily extract waxes, lipids, and nonhydroxylated terpenoids, which exhibit negligible electrochemical activity due to the lack of phenolic hydroxyl groups [34]. Although DCM and chloroform possess similar physicochemical properties, the lower anodic current observed for DCM fraction may be attributed to difference in distribution and relative abundance of redox active metabolite [34, 35].

**FIGURE 3:**
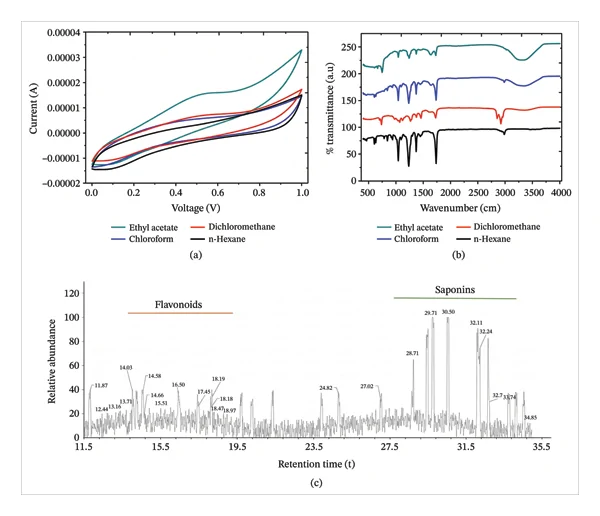
(a) n‐Hexane, DCM, chloroform, and ethyl acetate anodic peak currents (IPA) as voltammograms. (b) FT‐IR spectra of the four solvent extracts containing major functional groups (O‐H, C‐H, C=O, C=C, C‐O/C‐C) with the intensity change indicating the polarity of the solvent. (c) The LC‐MS chromatograms with the elution of flavonoids, saponins, triterpenoids, and lipid derivatives.

### 3.8. FT‐IR Analysis of Various Extracts

FT‐IR spectroscopy is widely used to identify the major functional groups present in plant‐derived extracts by measuring the vibrational transitions of chemical bonds. It provides rapid and reliable information on the chemical nature of phytoconstituents and helps in comparing compositional variations among extracts obtained using solvents of different polarities [36]. In this study, all four solvent extracts exhibited overall similar FT‐IR spectral profile, differing primarily in relative band intensities (Figure 3b). The spectra shows peaks at 3380–3450 cm^−1^ (O‐H stretch), 2985–2922 cm^−1^ (C‐H stretches), 1720–1740 cm^−1^ (C=O stretch), 1635 cm^−1^ (C=C band), 1440–1040 cm^−1^ (C‐O and C‐C vibrational), 1446 cm^−1^ (CH_2_ bending and CH_3_ deformation vibration), and 1043 cm^−1^ (C‐O stretch of alcohol/ether). There is a general O‐H peak located at 3380–3450 cm^−1^ (hydroxyl/phenolic groups) with the greatest intensity at the more polar fractions (ethyl acetate in particular) and the lowest intensity in an apolar n‐hexane. There are strong aliphatic C‐H stretches of 2985 cm^−1^ in chloroform and n‐hexane, and 2855–2922 cm^−1^ in the dichloromethane samples, which are absent in ethyl acetate indicating high lipid/aliphatic percentage of nonpolar extract. Each extract has a clear carbonyl C=O functional group at or around 1720–1740 cm^−1^, which is thecharacteristics of ester, ketone, and carboxylic acid. The intensity of this is relatively stronger in ethyl acetate and chloroform fractions compared to then‐hexane fraction, indicating a difference in the abundance of carbonyl containing constituents. Similarly, an aromatic/conjugated C=C band at 1635 cm^−1^ is present in ethyl acetate, which suggests more phenolic or unsaturated material. The C‐O and C‐C vibrational modes overlap in the region 1440–1040 cm^−1^ of all spectra: for example, there are bands at 1446 cm^−1^ (CH_2_ bending and CH_3_ deformation vibration) and 1043 cm^−1^ (C‐O stretch of alcohol/ether) observed in all fractions, but polar extracts have a little higher absorption.

### 3.9. LC‐MS Analysis of the Extracts

LC‐MS is an effective analytical method that is commonly used to separate, identify, and structurally characterize complex phytochemical compounds in plant extracts [37, 38]. By combining chromatographic resolution with high‐sensitivity mass detection, LC‐MS enables comprehensive profiling of both polar and nonpolar bioactive compounds, including flavonoids, saponins, triterpenoids, and lipid derivatives. The LC‐MS analysis was performed in this study to profile the phytochemical composition of the *Z. jujuba* leaves and provide the comprehensive identification of polar and nonpolar bioactive constituents (Figure 3c). Based on retention times (RT), deprotonated molecular ions [M – H]^−^, and characteristic fragmentation patterns, the detected compounds were tentatively identified in Table 1. The major eluted times were 11.87 and 28.71 min for the flavonoid compounds, whereas the saponins were detected between 14.03 and 16.50 min, as summarized in Table 1. Notable, flavonoids identified include vicenin II (RT = 11.87 min, MW = 594), spinosin (RT = 12.44 min, MW = 608), and kaempferol 3‐rutinoside (RT = 13.16 min, MW = 594). These compounds exhibited characteristic deprotonated molecular ions [M‐H]^−1^ and diagnostic fragments that confirmed their identity. The identification of vicenin 11 and kaempferol 3‐rutinoside is consistent with previous LC‐MS studies on *Ziziphus* species. These compounds, together with 3′,5′‐di‐C‐beta‐d glucosylphloretin and epiceanotic acid identified in this study, have been reported in *Z. jujuba* Mill. fruit [24, 39]. Furthermore, flavonoids such as vicenin II, kaempferol derivatives, and spinosin possess antioxidant activities, owing to their reducing capacity, free radical scavenging properties, thereby contributing to the antioxidant potential of *Z. jujuba* extracts [3]. Among the saponins, *Jujuboside A* (RT = 14.66, MW = 1206) was detected with high relative abundance and well‐resolved peak fragmentation peaks for these compounds (e.g., m/z 1187.4, 751.0). Additionally, a series of triterpenoids and ester derivatives, like alphitolic acid, epiceanothic acid, and glycerol ester 2, were also detected. According to the retention times of the MS/MS fragmentation patterns in support of the presence of the complex oleochemicals composition of *Z. jujuba* [3, 39], these triterpenoid saponins, such as jujuboside A and jujuboside B, together with triterpenoid acids including epiceanothic acid and alphitonic acid, have been associated with antioxidant and cytoprotective activities in earlier studies [40]. Moreover, a flavonoid ester 3′,5′‐di‐C‐beta‐d glucosylphloretin was identified (RT = 28.71 min, MW = 598), with fragment ions at m/z 506.90 and 477.4. Other compounds were also found, such as linoleic acid derivatives (RT = 32.11 min, MW = 298) and linoleic acid (RT = 32.55 min, MW = 280), and jujuboside B (RT = 16.50 min, MW = 1044). The presence of linoleic acid and its derivatives further demonstrate the presence of lipid‐derived phytochemicals that may contribute to the biological activity of the *Z. jujuba* extract. Similarly, a diverse phytochemical profile, consisting flavonoids, saponins, organic acids, and triterpenoids, has been reported in *Z. jujuba* tissues [41]. These compounds demonstrate the diversity of bioactive constituents present in *Z. jujuba* leaves. The results are also quite consistent with the earlier reported LC‐MS investigations on *Z. jujuba* [42, 43], which reported the same elution behavior, molecular ions, and fragmentation patterns of flavonoids, saponins, and triterpenoids. Together, these consistent observations confirmed the reliability of the LC‐MS for comprehensive phytochemical profiling of this species.

**TABLE 1:** Tentative LC‐MS identifications of phytochemical constituents in *Ziziphus jujuba* leaves extracts.

Retention time (min)	Molecular weight (MW)	[M–H]^−^ (m/z)	Fragment ions (m/z)	Chemicals	Class
11.87	594	592.8	412.6, 352.7	Vicenin II	Flavonoid
12.44	608	606.7	486.8427.2, 366.9, 118.8	Spinosin	Flavonoid (C‐Glycoside)
13.16	594	592.9	447.2, 340.8, 284.5	Kaempferol 3‐rutinoside	Flavonoid
13.71	784	782.8	606.5, 193.2, 178.8	6″‐Feruloylspinosin	Flavonoid
14.03	942	941.8	470.8, 416.7, 325.4	Jujuba saponin V	Saponin
14.58	1119	1117.8	836.9213.6	—	—
14.66	1206	1205.1	1187.4, 751.0	Jujuboside A	Saponin
16.5	1044	1043.1	910.9, 310.6	Jujuboside B	Saponin
18.18	596	594.9	577.1, 559.4, 475.4	6,8‐Diglucopyranosyl 40,5,7 trihydroxyfavanone	Flavonoid
18.19	477	475.7	953.2	—	
18.47	472	470.9	452.8, 393.1	Alphitolic acid	Triterpenoid
18.97	572	570.8	411.3390.5314.6254.9240.9152.7	—	
28.71	598	596.9	506.9, 477.4	3′,5′‐Di‐C‐beta‐d glucosylphloretin	Flavonoid
29.71	486	485	471.4, 441.0, 422.8	Epiceanothic acid	Triterpenoid
30.5	296	294.9	276.8, 194.8		—
32.11	298	296.8	278.9, 168.9	Linoleic acid derivative	lipid derivative
32.24	834	833.2	553.1	Glycerol ester 2	Ester
32.55	280	278.9	260.8, 70.6,58.7	Linoleic acid	Lipid

## 4. Conclusion

The study shows that the extraction yield, phytochemical distribution, and electrochemical behavior of extracts from *Z. jujuba* leaves are significantly influenced by the solvents polarity. The greatest crude yield (3.00 ± 0.04 g; 6.00 ± 0.07% w/w) was obtained by ethanol, demonstrating its efficiency in the broad‐spectrum extraction of plant metabolites. Solvent polarity‐guided enrichment of bioactive compounds through successive liquid–liquid partitioning. The ethyl acetate friction had the highest levels of flavonoid (*A* = 0.711 at 415 nm) and saponins (*A* = 0.330 at 460 nm) according to UV‐visible spectrophotometric analysis. In comparison, the chloroform showed flavonoid absorbance of *A* = 0.602 and saponin absorbance of *A* = 0.1275, while the DCM fraction exhibited flavonoid and saponin absorbance of *A* = 0.489 and *A* = 0.210, respectively, and the n‐hexane demonstrated the lowest values, that is, *A* = 0.332 for flavonoids and *A* = 0.125 for saponins. These findings were quantitatively supported by TLC profiling, which showed that there was more phytochemical diversity in the midpolarity fractions as the number of distinct bands increased from n‐hexane (three bands; Rf = 0.24–0.88), DCM (five bands; Rf = 0.18–0.83), chloroform (six bands; Rf = 0.20–0.87), and ethyl acetate (seven bands; Rf = 0.15–0.89). All the findings demonstrate that the most effective recovery of flavonoids and saponins bioactive substances from *Z. jujuba* leaves is achieved with midpolarity solvent, especially ethyl acetate. Solvent polarity also has a direct impact on extraction efficiency, phytochemical diversity, and electrochemical activity.

## Funding

This study was supported by SASOL‐NRF, 138626.

## Conflicts of Interest

The authors declare no conflicts of interest.

## Data Availability

The data that support the findings of this study are available within the article. No additional datasets were generated or analyzed during the current study.
